# Cryptic but Deadly: A Serious Killer in Patients with Advanced Liver Disease that Should Not Be Missed

**DOI:** 10.7759/cureus.1976

**Published:** 2017-12-21

**Authors:** Jeffrey Berinstein, Alisa Likhitsup, Sai Charan Vedula, Hari Conjeevaram

**Affiliations:** 1 Internal Medicine, University of Michigan; 2 Division of Gastroenterology and Hepatology, University of Michigan; 3 M S Ramaiah Hospital, M S Ramaiah Medical College; 4 Division of Gastroenterology and Hepatologoy, University of Michigan

**Keywords:** cirrhosis, cryptococcus, spontaneous peritonitis, hepatocellular carcinoma, autoimmune hepatitis, immunosuppression, spontaneous fungal peritonitis, infection, fungal infection, invasive fungal infection

## Abstract

Cryptococcal peritonitis is an under-recognized disease that is an important cause of mortality in end-stage liver disease. We report a 43-year old male with decompensated cirrhosis secondary to refractory autoimmune hepatitis on immunosuppression with hepatocellular carcinoma who developed cryptococcal peritonitis. The patient subsequently developed ischemic bowel and multisystem organ failure secondary to abdominal compartment syndrome, leading to rapid deterioration and death. Frequently, these patients experience delays in diagnosis and treatment, which leads to a rapid and high mortality. This case report synthesizes data regarding the optimal approach for screening and managing patients with cryptococcal peritonitis and proposes a pathogenic mechanism of mortality with implications for improved treatment in the future.

## Introduction

Spontaneous peritonitis (SP) is the most common infectious complication among patients with cirrhosis and ascites, occurring in 10%-20% of these patients [1]. SP is typically caused by bacterial infections (spontaneous bacterial peritonitis or SBP); however, spontaneous peritonitis due to fungal infection (spontaneous fungal peritonitis or SFP) can rarely occur (3%-4% of patients with SP) [2-3]. The Candida species is the most common causative pathogen, occurring in 60%-100% cases of SFP [2-4]. The Cryptococcus species has also been identified as a clinically important cause of SFP that occurs much less frequently [3]. Although cryptococcal infections are most often associated with the human immunodeficiency virus (HIV) infection, the cryptococcal disease has also been reported in patients with other intrinsic or extrinsic immunosuppression, notably in patients with advanced liver disease [5].

## Case presentation

A 43-year-old male with a history of decompensated cirrhosis secondary to refractory autoimmune hepatitis with hepatocellular carcinoma on triple therapy, with budesonide 3 mg, prednisone 5 mg, and azathioprine 50 mg, was transferred from an outside hospital for septic shock secondary to a complicated enterococcus urinary tract infection (UTI). On admission, the patient’s model for end-stage liver disease (MELD)-Na was 32 (creatinine: 1.3 mg/dL, total bilirubin: 21.2 mg/dL, international normalized ratio (INR): 1.7, and Na: 123 mmol/L). Initial vital signs were temperature: 35.1℃, heart rate: 89 beats per minute (BPM), respiratory rate: 18 BPM, blood pressure: 96/48 mmHg, and peripheral capillary oxygen saturation (SpO2): 98% on room air. The physical exam was significant for a chronically ill-appearing obese male in no acute distress. The patient was alert, oriented, and had bilateral asterixis. His skin was visibly jaundiced. A fluid wave and shifting dullness were appreciated on the abdominal exam; however, the patient was without tense ascites, tenderness, or peritoneal signs. The patient demonstrated 2+ lower extremities edema to knees bilaterally. Initial paracentesis on the day prior to transfer was negative for SP (white blood cells (WBC): 80 cells/cmm), as was a subsequent paracentesis (WBC 64 cells/cmm with 42% polymorphonuclear cells (PMNs)) performed 12 days later, following the completion of antibiotics for his complicated UTI. On hospital day 13, the patient became hypotensive and complained of severe abdominal pain. He was transferred to the intensive care unit (ICU) for the escalation of care. Given the chronic immunosuppression, both bacterial and fungal pathogens were investigated. Fungitell B-D-Glucan returned positive (255 pg/mL). Serum cryptococcal antigen (CrAg) returned positive (titer: 1:20). Subsequently, the ascitic fluid culture from the previous day grew yeast, which eventually speciated into Cryptococcus neoformans. Broad-spectrum antibiotics (vancomycin, cefepime, and metronidazole) were initiated along with fluconazole for antifungal coverage. The lumbar puncture was negative (WBC: 4 cells/cmm, protein: 46 mg/dL, glucose: 70 mg/dL, and cerebral spinal fluid (CrAg) was negative). Computed tomography (CT) thorax was negative for lung involvement. The urinary bladder pressure (UBP) measurement was 29 mmHg, which was consistent with intra-abdominal hypertension (IAH) as well as abdominal compartment syndrome (ACS) (defined as UBP > 20 mmHg). The patient underwent a therapeutic paracentesis with the removal of 6.5 L of fluid with an improvement in UBP to 10 mmHg. The ascitic fluid analysis was consistent with spontaneous peritonitis (WBC: 654 cells/cmm; PMNs: 61%). Fluconazole was switched to liposomal amphotericin B and flucytosine.

**Figure 1:**
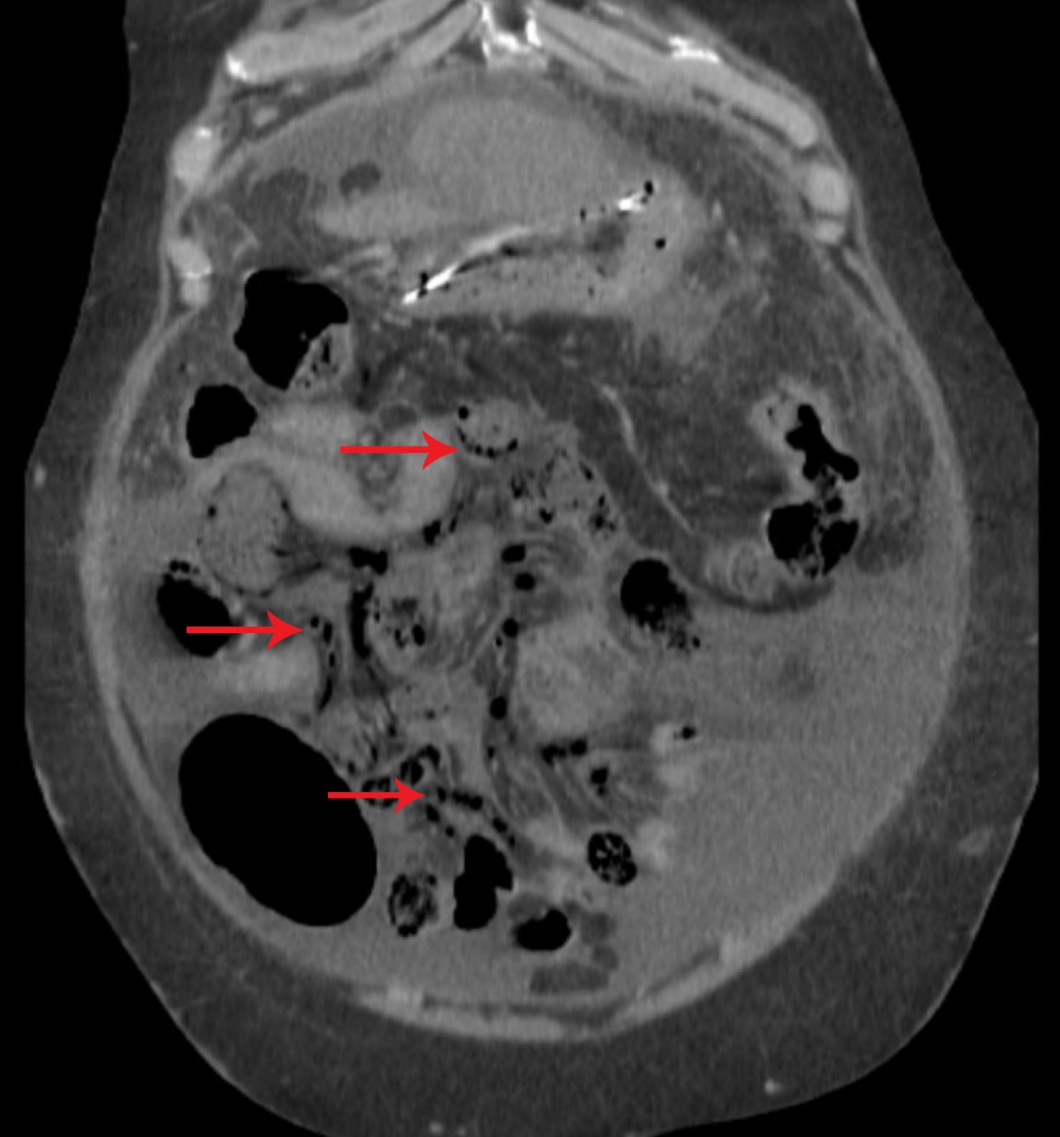
CT abdomen and pelvis demonstrating extensive mesenteric gas (red arrows) concerning for bowel ischemia in the setting of cryptococcal peritonitis induced abdominal compartment syndrome in a patient with advanced liver disease CT: computed tomography

Subsequently, the patient developed acute oliguric renal failure, which was felt to be due to poor renal perfusion in the setting of abdominal compartment syndrome. He was briefly intubated due to respiratory failure from volume overload at which point continuous renal replacement therapy was initiated. He required repeat paracentesis every two to four days due to abdominal pain and concern for worsening IAH. Unfortunately, 15 days after being transferred to the ICU, the patient developed severe abdominal pain out of proportion to the physical exam and became hypotensive, requiring sequential reinitiation of three vasopressors. A CT abdomen was performed, demonstrating extensive venous mesenteric gas, concerning for bowel ischemia (Figure 1). General surgery was consulted, who felt the intraoperative risks were too high to offer surgical intervention. The patient was transitioned to comfort care and expired shortly after.

## Discussion

Cryptococcus is an encapsulated, opportunistic yeast found ubiquitously in soil frequented by birds, especially pigeons and chickens [5]. Cryptococcal infections are most often associated with HIV infections; however, advanced liver diseases are increasingly being identified as a clinically important risk factor as well. Clinical manifestations differ in patients, depending on the underlying illness. The central nervous system (CNS) and pulmonary infections are the most common clinical manifestations in HIV patients infected with Cryptococcus [5], whereas peritonitis is the most common clinical manifestation of patients with end-stage liver disease (ESLD) (45.4%) [6]. Specifically, ESLD patients with high MELD scores (mean 34) are at the highest risk for cryptococcal peritonitis [4]. Additional risk factors for acquiring cryptococcal peritonitis in patients with advanced liver diseases include high creatinine levels [7], recent antibiotic use (eg. SBP prophylaxis) [4,8], high number of invasive procedures [2], and prolonged hospital stays [2-3]. Prior to developing cryptococcal peritonitis, our patient had a prolonged hospital stay, multiple paracenteses, as well as intermittent Foley placement, broad-spectrum antibiotic administration, and elevated creatinine.

SFP is often difficult to diagnose, owing to lack of clinical awareness, lack of characteristic symptoms, modest ascitic fluid pleocytosis, and slow culture turnaround time. In a case series of patients with cirrhosis and cryptococcal peritonitis, the median ascitic fluid WBC count was 340 WBC/cmm with 38% having less than 200 WBC/cmm [6]. The median time to positive ascitic fluid culture for Cryptococcus was six days [6]. Given the slow culture turnaround time and the modest ascitic fluid pleocytosis, surrogate screening markers for fungal infection should be considered. These include ascitic fluid lactate dehydrogenase (LDH) [2], serum and ascitic Fungitell (Associates of Cape Cod, Massachusetts, USA) β-d-glucan [8], and serum and ascitic CrAg [6].

Several studies have demonstrated a significantly higher 30-day and overall mortality in patients with SFP compared to SBP [2,4]. A recent study demonstrated a significantly higher 90-day and more rapid mortality in patients with cryptococcal infection and ESLD compared to patients without ESLD (80% 90-day mortality and a six-day median survival compared to 18% 90-day mortality and 14-day median survival) [9]. Interestingly, not much is known about the cause of mortality in patients with ESLD and cryptococcal peritonitis. The predominant factor leading to poor outcomes in these patients is plausibly a delay in diagnosis and treatment; however, the development of abdominal compartment syndrome may be an under-recognized and under-treated entity contributing to mortality in these patients. It is known that that cryptococcal meningitis leads to death through increased intracranial pressure (ICP) [10]. The proposed mechanism of increased ICP in cryptococcal meningitis is that increased vascular permeability occurs secondary to cytokine-induced inflammation, leading to clogging of arachnoid villi with a fungal antigen and impaired resorption of CSF, leading to fluid accumulation [10]. Perhaps, mortality in patients with cryptococcal peritonitis occurs via a similar mechanism in which inflammation leads to reduced peritoneal fluid reabsorption; the result of which is increased intra-abdominal hypertension and abdominal compartment syndrome, leading to multiorgan failure, as seen in our patient. Vigilant intra-abdominal pressure monitoring, early surgical consultation, and serial therapeutic paracentesis should be considered, in addition to antifungal therapy, in these patients, to minimize the risk of complications and rapid progression to mortality.

## Conclusions

SFP is an often under-recognized and clinically important infectious complication seen in patients with an underlying advanced liver disease with the following risk factors: high creatinine, prior antibiotic use, recent invasive procedures, and prolonged hospital stays. While the causative organism of SFP is most commonly Candida, Cryptococcus has emerged as an underappreciated and often rapidly fatal clinical entity. SFP is often difficult to diagnose, owing to a lack of clinical awareness, a lack of characteristic symptoms, modest ascitic fluid pleocytosis, and slow culture turnaround time. It is for this reason that surrogate screening tests, such as ascitic fluid LDH, serum and ascitic Fungitell β-D-Glucan, and serum and ascitic CrAg should be considered. In patients with negative culture or patients who are unresponsive to standard antibiotic therapy, empiric antifungal agents should be considered in all patients with ESLD. In all patients diagnosed with cryptococcal peritonitis, intra-abdominal pressure monitoring, early surgical evaluation, and serial therapeutic paracentesis should be considered to recognize and manage intra-abdominal hypertension early and reduce the risks of developing multiorgan failure secondary to abdominal compartment syndrome.
